# Influencing factors of falls among older adults in Chinese retirement institutions: A systematic review and meta-analysis

**DOI:** 10.1371/journal.pone.0296348

**Published:** 2023-12-27

**Authors:** Xiaoxing Huang, Yunlan Jiang, Yaxin Liu, Liyin Shen, Jing Pan, Yue Zhang

**Affiliations:** 1 College of Nursing, Chengdu University of Traditional Chinese Medicine, Chengdu City, Sichuan Province, China; 2 Hospital of Chengdu University of Traditional Chinese Medicine, Chengdu City, Sichuan Province, China; Dayeh University, TAIWAN

## Abstract

**Background:**

The incidence of falling has always been high among the elderly, and it was easy to cause injuries to the elderly and seriously affect their quality of life. There were many studies have been conducted on risk factors affecting the fall of the elderly, but the results widely, retirement institutions as a gathering place for the elderly, there was currently no comprehensive analysis of the factors related to elderly falls in pension institutions. This study aimed to explore the influencing factors of falls among older adults in Chinese nursing homes.

**Methods:**

Chinese and English databases were searched for literature published from database inception to 5 April 2023 on the influencing factors of falls among older adults in Chinese nursing homes. Two reviewers independently screened articles, extracted data, and assessed the quality of the included studies. Meta-analysis was performed using RevMan 5.4 software.

**Results:**

Eleven studies involving 3503 participants were included in the meta-analysis. The pooled estimate of falls among older adults in Chinese nursing homes was 32% [95% confidence interval (95%CI) (24.0%, 39.0%)]. The main influencing factors for falls among older adults in Chinese nursing homes were age (Odds Ratio (OR) = 1.53), gender (OR = 5.50), visual impairment (OR = 2.30), sedative-hypnotics (OR = 2.36), fear of falling (OR = 2.95), hypertension (OR = 3.72), static balance (OR = 2.02), three or more chronic diseases (OR = 5.63), cognitive status (OR = 2.64), walking aid use (OR = 1.98), fall-related chronic diseases (OR = 2.48), self-awareness of abilities (OR = 2.43), and frequent reminders for fall prevention (OR = 0.10).

**Conclusion:**

Falls among older adults in Chinese nursing homes were common, and there were many influencing factors. Timely screening and intervention should be implemented to reduce the adverse consequences of falls on older adults.

**Trial registration:**

**Registration number:** CRD42023421099.

## 1 Introduction

The results of China’s seventh national census showed that the population of elderly people aged 60 and above has reached 264 million, accounting for 18.7% of the total population [1]. With the increasing number of only children and the majority of them working outside their hometowns, home-based elderly care can no longer meet the social demands of contemporary society. As a result, more and more elderly people are choosing to live in elderly care institutions [2, 3].

Falling is an unintentional act of falling to the ground or a lower surface, but does not include violence, loss of consciousness, paralysis, or seizures as causes [4]. As the most common injurious behavior, it was a major health problem faced by older people worldwide. It has the highest incidence and mortality rates in accidental injuries among older people, not only causing physical and mental harm but also seriously affecting their quality of life. Falling also imposed heavy economic and care burdens on families and society [5–7].

Research has shown that the incidence of falls among elderly people living in elderly care institutions is higher, with a rate of 30% to 50%, which was three times higher than that of elderly people in the community. The annual incidence rate of falls in people aged 65 and above exceeded 50%, and 4% to 15% of falls result in serious injuries [8, 9]. Some studies conducted meta-analysis on fall-related factors of the elderly suffering from different diseases such as hypertension and stroke, or conducted quantitative and comprehensive analysis on the overall fall rate of the elderly in China. At the same time, due to the influence of research location and social environment, the fall rate and related influencing factors obtained in the existing literature are different, and there is a lack of a comprehensive quantitative analysis. No studies on the influencing factors of falls among older adults in Chinese retirement institution have been found. Therefore, it was necessary to comprehensively understand the influencing factors of falls among elderly people in Chinese elderly care institutions and provide targeted care. The aim of this study is to explore the influencing factors of falls among elderly people in Chinese elderly care institutions through a meta-analysis and provide theoretical basis for the prevention of falls among elderly people in elderly care institutions.

## 2 Materials and methods

### 2.1. Registration

The protocol of this review was registered in the International Prospective Register of Systematic Overview (PROSPERO) database (https://www.crd.york.ac.uk/PROSPERO/), registration number: CRD42023421099.

### 2.2. Ethics

Ethics approval is not required in Meta-analysis.

### 2.3 Literature search strategy

The literature search strategy included comprehensive searches of several databases, including Chinese Biomedical Literature Database (CBM, http://www.sinomed.ac.cn/index.jsp), China National Knowledge Infrastructure (CNKI, https://www.cnki.net/), VIP Database (http://lib.cqvip.com/) and Wanfang Database (https://new.wanfangdata.com.cn/index.html), Embase, Web of Science, The Cochrane Library, and PubMed, up to 5 April 2023, for cross-sectional studies, cohort studies, and case-control studies related to the influencing factors of falls among elderly residents in Chinese retirement institutions. In addition, relevant references from the included articles were manually searched and added. The English search strategy was determined as follows: (the aged OR elder people OR senior citizens OR old folks OR the elderly OR old people) AND (senior housing OR senior center OR residential aged care facility OR old age homes OR nursing home residents OR old-age care institutions OR the old folk′s homes OR retirement home OR rest home) AND (falls OR falling OR accidental fall OR slip and fall) AND (risk factor OR predictor OR influencing factor OR protective factor OR promotive factor OR correlate).

### 2.4 Inclusion and exclusion criteria for literature selection

#### 2.4.1 Inclusion criteria for literature selection

(1) Study population: Elderly individuals aged 60 years and above; (2) Study topic: Falls and their influencing factors among elderly residents in Chinese nursing homes; (3) Study design: Cross-sectional studies, case-control studies, or cohort studies; (4) Articles must report the Odds Ratios (ORs) and 95% Confidence Intervals (CIs) of relevant influencing factors; (5) Articles must be published in either Chinese or English and available online.

#### 2.4.2 Exclusion criteria for literature selection

(1) Studies with a quality score of less than 4 points, indicating low-quality research; (2) Studies for which the full text or sufficient data cannot be obtained; (3) Duplicate publications of the same study; (4) Conference abstracts, systematic reviews, animal experiments, case reports, experience summaries, or other types of non-original research articles.

### 2.5 Article screening and data extraction

All searched literature was imported into the Endnote X9 software, and two researchers independently conducted title and abstract screening, as well as full-text reading to perform the initial and secondary screening of literature and extract relevant information. In case of any discrepancies, they would be resolved through discussion or consultation with a third party. The information extracted will include the author, year of publication, study location, study design, sample size, rate of falling, relevant factors, quality score, and other relevant information.

### 2.6 Quality assessment

The Newcastle-Ottawa scale (NOS) [10] is used to evaluate case-control and cohort studies. It consists of eight items, with scores ranging from7 to 9 indicating high-quality literature, 5 to 6 indicating moderate-quality literature, and 0 to 4 indicating low-quality literature. The Agency for Healthcare Research and Quality (AHRQ) [11] in the United States uses a similar scale to evaluate cross-sectional studies, with total scores ranging from 8 to 11 indicating high-quality studies, 4 to 7 indicating moderate-quality, and 0 to 3 indicating low-quality. Methodological quality evaluation is independently conducted by two evaluators. In the event of disagreements, they can resolve them through discussion or have a third researcher make the final decision.

### 2.7 Statistical analysis

Descriptive analysis was used in most sections to report relevant content, and the statistical software package RevMan 5.4 was used to analyze quantitative data extracted from each study. OR values and their 95% CIs were used for statistical combination, and the χ^2^ test (with a significance level of α = 0.1) combined with I^2^ value was used to determine the degree of heterogeneity. If P≤0.1 and I^2^>50%, a random effects model was used for meta-analysis; otherwise, a fixed effects model was used for meta-analysis. Descriptive analysis was used for influencing factors that were not suitable for meta-analysis. Sensitivity analysis was conducted for each influencing factor by transforming different effect models. If the number of studies was ≥10, Egger’s test and Begg’s test were used to evaluate publication bias in the literature.

## 3 Results

### 3.1 Literature search results

**Fig 1:**
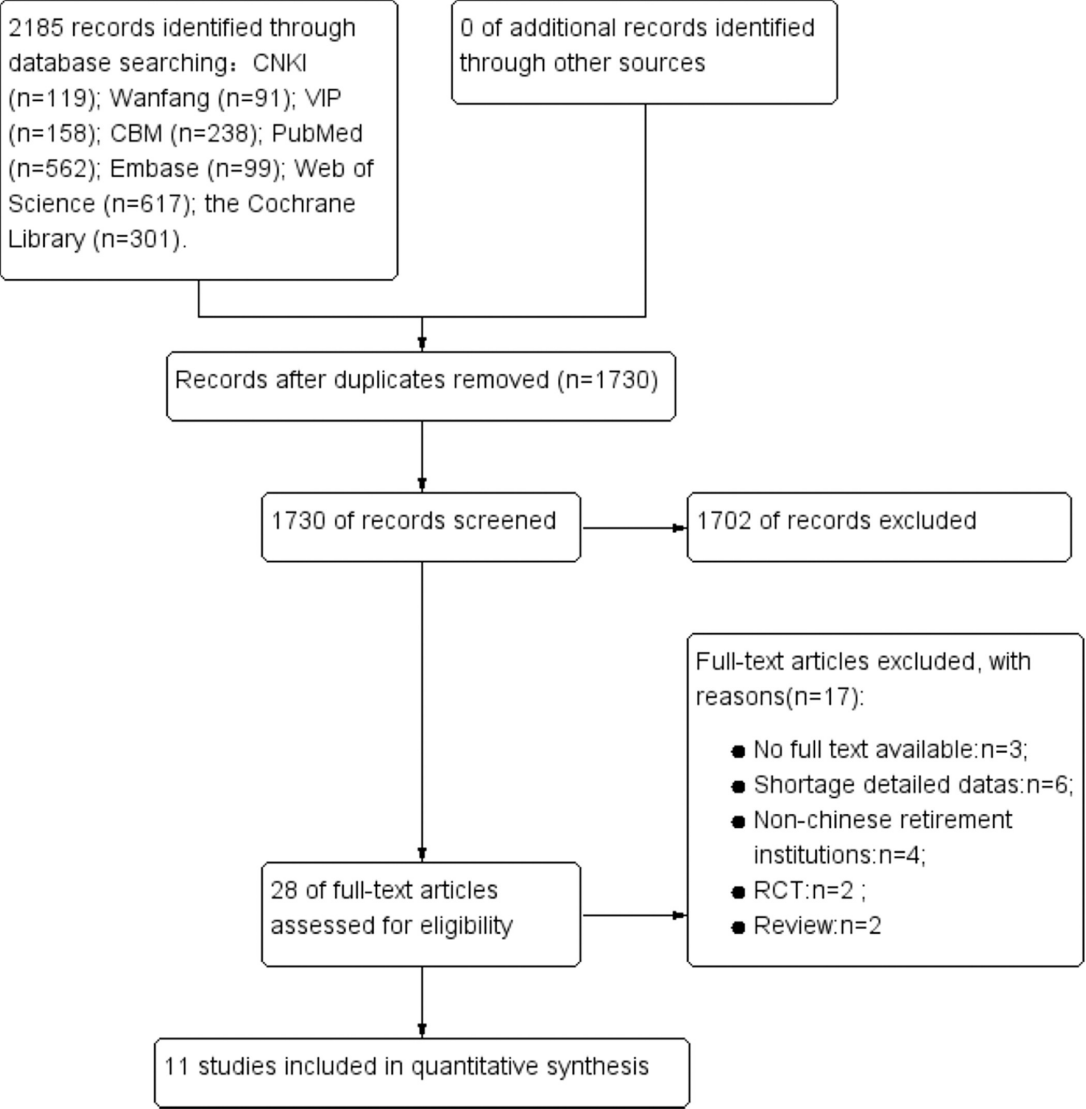


A total of 2185 literature sources were initially retrieved, including 606 in Chinese and 1579 in English. Literature sources were then screened step by step, and 11 studies [12–22] were ultimately included in the present study. The literature screening process is shown in **Fig 1.**

### 3.2 Characteristics of included literature and results of literature quality assessment

A total of 11 literature sources with a combined sample size of 3503 were included in the analysis, with 1126 cases in the fall group and 2377 cases in the non-fall group. The NOS scores for the case-control and cohort studies were both ≥5 points, and the AHRQ scores for the cross-sectional studies were all ≥4 points, all studies included in the analysis were of medium to high quality, indicating that the literature quality met the requirements. The main information extracted from the literature sources is shown in **Table 1.**

**Table 1:** Characteristics of included studies and quality assessment results.

First authors	Year	Region	Study design	The survey time periods	The time periods for falls	Fall example	Non-fall example	Total sample size	Fall rate (%)	Factors	Quality assessment tool	Quality score
Li Cuizha	2022	Kunming City	Cross-sectional study	May to July 2021	——	82	141	223	36.77	adn	AHRQ	7
Feng Wenting	2022	Xinyang,	Cross-sectional study	April to October 2020	In the past year	107	324	431	24.83	abcfjklmno	AHRQ	8
Lin Shuang	2021	Shenyang,	Cross-sectional study	July to September 2018	In the past year	114	569	683	16.70	cjo	AHRQ	9
Hu Huiju	2021	Tangshan,	Cross-sectional study	August to November 2020	In the past year	223	267	490	45.50	aelp	AHRQ	8
Cheng Xiao	2020	Chenzhou	Cross-sectional study	October to December 2018	In the past year	41	79	120	34.16	cghi	AHRQ	6
Zhang L	2019	Xiamen	Cross-sectional study	June to September 2016	In the past year	69	149	218	31.65	dgikl	AHRQ	8
Liang Danyan	2017	Hohhot	Cross-sectional study	——	August 2015 to August 2016	27	63	90	30.00	bfmp	AHRQ	7
Zhao Ming	2016	Hangzhou	Cross-sectional study	——	November 2012 to October 2013	48	322	370	12.97	ae	AHRQ	6
Zhang Yu	2016	Urumqi	Cross-sectional study	June to December 2015	In the past year	232	264	496	46.77	cdgnq	AHRQ	7
Chen Yang	2014	Nanjing City	Cross-sectional study	October 2012 to January 2013	In the past year	29	45	74	39.19	dkq	AHRQ	5
Liu Yongyi	2002	Beijing	1:1 Case-control study	——	Within the past 18 months.	154	154	308	——	dfgh	NOS	7

Note: "——" indicates not applicable; AHRQ = Agency for Healthcare Research and Quality; NOS = Newcastle-Ottawa Scale; a = age; b = gender; c = visual impairment; d = sedative-hypnotic medication; e = reduced activity due to fear of falling; f = hypertension; g = static balance; h = three or more chronic conditions; i = cognitive status; j = mobility aid; k = fall-related chronic conditions; l = activities of daily living; m = indoor lighting; n = regular physical exercise; o = realistic self-perception; p = frequently reminded to prevent falls; q = sleep.

### 3.3 The fall rate of elderly residents in Chinese retirement institutions

**Fig 2:**
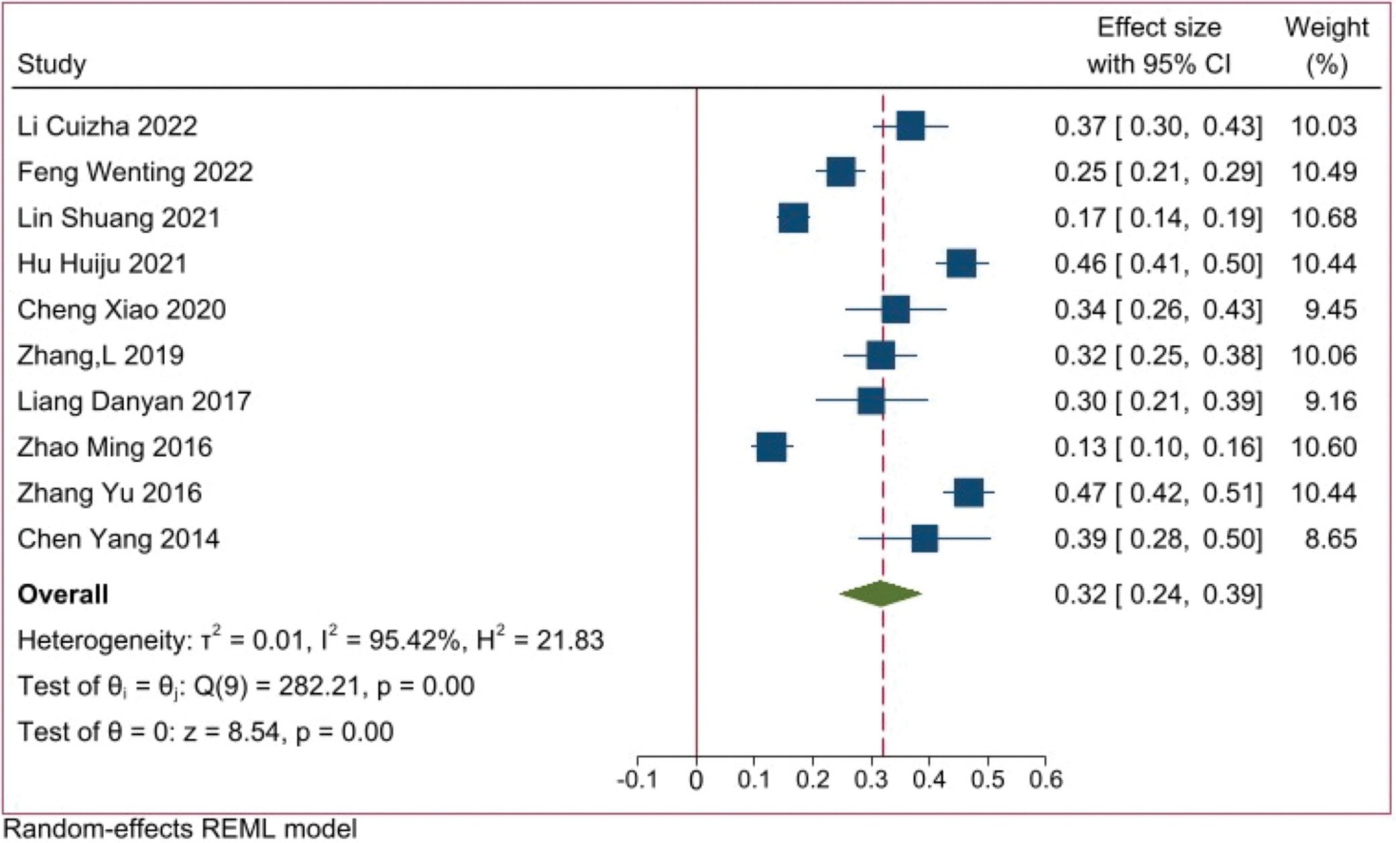


The final inclusion in the literature review consisted of a one-to-one case-control study that did not involve fall rates. Therefore, a quantitative synthesis was conducted specifically for the fall rates mentioned in the remaining ten studies. The analysis results showed that the fall rate of elderly residents in Chinese nursing homes was 32% [95%CI (0.24, 0.39), P < 0.001] (**Fig 2**).

### 3.4 Influencing factors and results

#### 3.4.1 Meta-analysis results

**Fig 3:**
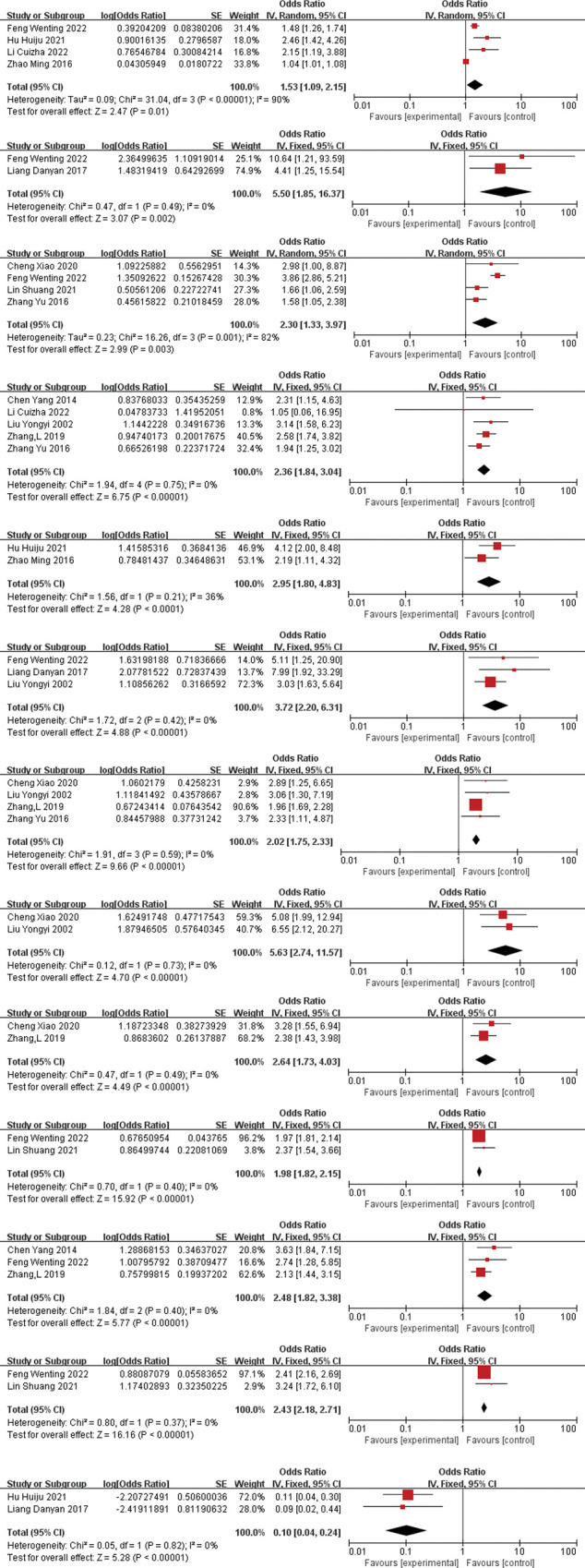


Two or more study reporting the same influencing factors were combined using RevMan5.4 software, and 17 influencing factors related to falls among elderly residents in nursing homes were included in the quantitative analysis. The meta-analysis results showed that age (OR = 1.53, 95%CI = 1.09–2.15), gender(OR = 5.50, 95%CI = 1.85–16,37), visual impairment(OR = 2.30,95%CI = 1.33–3.97), sedatives-hypnotics(OR = 2.36,95%CI = 1.84–3.04, fear of falling(OR = 2.95, 95%CI = 1.80–4.83), hypertension(OR = 3.72, 95%CI = 2.20–6.31), postural balance(OR = 2.02, 95%CI = 1.75–2.33), three or more chronic diseases(OR = 5.63, 95%CI = 2.74–11.57), cognitive status(OR = 2.63, 95%CI = 1.73–4.03), walking aids(OR = 1.98, 95%CI = 1.82–2.15), fall-related chronic diseases(OR = 2.48, 95%CI = 1.82–3.38), self-awareness of abilities(OR = 2.43, 95%CI = 2.18–2.71), being frequently reminded to prevent falls(OR = 0.10, 95%CI = 0.04–0.24), were statistically significant factors related to falls among elderly residents in Chinese nursing homes(P<0.05). However, indoor lighting, regular physical exercise, and sleep were not significantly associated with falls. Please refer to **Table 2** for details. And the forest plot for 13 influencing factors refer to **Fig 3** (From Fig 3A to 3M).

**Table 2:** Meta-analysis and sensitivity analysis results of factors influencing falls among elderly residents in Chinese nursing homes.

Influencing Factors	Number of included studies	Heterogeneity test results		Meta-analysis results		Sensitivity analysis
		*I*^2^(%)	*P*	effect model	*OR* (95%*CI*)	*OR* (95%*CI*)
Age (≥60 years old)	4	90	<0.00001	random	1.53 (1.09, 2.15) *	1.07 (1.03, 1.10)
gender	2	0	0.49	fixed	5.50 (1.85,16.37) *	5.50 (1.85,16.37)
visual impairment	4	82	0.001	random	2.30 (1.33, 3.97) *	2.52 (2.05, 3.11)
sedative-hypnotics use	5	0	0.75	fixed	2.36 (1.84, 3.04) *	2.36 (1.84, 3.04)
reduced activity due to fear of falling	2	36	0.21	fixed	2.95 (1.80, 4.83) *	2.97 (1.60, 5.51)
hypertension	3	0	0.42	fixed	3.72 (2.20, 6.31) *	3.72 (2.20, 6.31)
postural balance	4	0	0.59	fixed	2.02 (1.75, 2.33) *	2.02 (1.75, 2.33)
having three or more chronic diseases	2	0	0.73	fixed	5.63(2.74, 11.57) *	5.63(2.74, 11.57)
cognitive status	2	0	0.49	fixed	2.64 (1.73, 4.03) *	2.64 (1.73, 4.03)
walking aids	2	0	0.4	fixed	1.98 (1.82, 2.15) *	1.98 (1.82, 2.15)
fall-related chronic diseases	3	0	0.4	fixed	2.48 (1.82, 3.38) *	2.48 (1.82, 3.38)
activities of daily living	3	88	0.0003	random	1.26 (0.38, 4.16)	1.64 (1.23, 2.18)
indoor lighting	2	95	<0.0001	random	1.25 (0.02, 94.64)	6.72 (3.70, 12.22)
regular physical exercise	3	85	0.001	random	1.38 (0.44, 4.33)	1.24 (0.80, 1.92)
self-awareness of abilities	2	0	0.37	fixed	2.43 (2.18, 2.71) *	2.43 (2.18, 2.71)
being frequently reminded to prevent falls	2	0	0.82	fixed	0.10 (0.04, 0.24) *	0.10 (0.04, 0.24)
sleep	2	86	0.009	random	0.92 (0.27, 3.06)	1.36 (0.99, 1.85)

Note

*P<0.05.

#### 3.4.2 Descriptive analysis

The studies [12–14, 17, 18, 22] have identified that the use of analgesics, arthritis, osteoporosis, dizziness, poor overall health assessment, no spouse, post-stroke sequelae, sensory loss, wearing slippers, drinking alcohol, and taking four or more medications are factors influencing falls among elderly residents in nursing homes. However, due to the limited number of studies on these influencing factors, only qualitative descriptions can be provided.

#### 3.4.3 Sensitivity and publication bias analysis

According to the sensitivity analysis conducted using the transformation model, the consistency of all influencing factors was found to be stable, indicating that the results are stable and reliable (**Table 2**). Since the number of studies included for each influencing factor is less than 10, and the significance of publication bias for the literature on fall rates is low, no publication bias analysis was conducted.

## 4 Discussion

After comprehensive search and strict literature screening, this Meta-analysis included 11 articles consisting of 10 cross-sectional studies and 1 case-control study, all of which clearly stated the inclusion and exclusion criteria of the investigated subjects and the factors influencing falls. The quality of the literature meets the requirements, and the statistical methods used are correct. Therefore, the meta-analysis has high credibility.

### 4.1 The incidence of falls among elderly residents in Chinese nursing homes is relatively high

A total of 11 Chinese literature on factors influencing falls among elderly residents in nursing homes were included in this study, comprising 3,503 research subjects from 11 provinces, cities, and autonomous regions in China. Meta-analysis results showed that the incidence of falls among elderly residents in Chinese nursing homes was 32% [95% CI (24%, 39%), P < 0.001].

### 4.2 Analysis of factors influencing falls among elderly residents in Chinese nursing homes

#### 4.2.1 General factors

The results of this study show that age, gender, and poor static balance are risk factors for falls among elderly residents in nursing homes. As the body’s physiological functions decline and organs age with increasing age, reaction time lengthens and various balance abilities deteriorate, leading to a higher incidence of falls and more severe injuries after falls [23]. Females experience a decline in estrogen levels after menopause, which increases the risk of osteoporosis, while female muscle strength and physique are relatively poor compared to males [24]. This is consistent with the results of previous studies conducted by Yao Yuhua et al. [25], which found that older women have a higher risk of falls. An unstable gait can easily lead to a loss of balance and falls, which is consistent with the results of Wang Liancheng’s study [26]. Therefore, as a place where elderly people live together, nursing homes should strengthen the care of high-risk groups for falls and regularly provide rehabilitation training targeted at improving balance ability to prevent falls.

#### 4.2.2 Disease-related factors

As the elderly population ages, the risk of developing multiple chronic diseases gradually increases. Studies have shown that the prevalence of chronic diseases among elderly people in China is 43.6% [27]. The results of this study showed that the risk factors for falls among elderly people in Chinese nursing homes include having a fall-related chronic disease (OR = 2.48) and having three or more chronic diseases (OR = 5.63). However, due to limitations in the available data from the literature included in this study, specific types of chronic diseases were not analized. The study shows that visual impairment (OR = 2.30) is a risk factor for falls, and factors affecting vision include cataracts, glaucoma, retinal vascular disease, age-related macular degeneration, and diabetic retinopathy [28]. Compared to adults with normal vision, adults with visual impairment are 1.7 times more likely to fall and 1.9 times more likely to fall multiple times [29]. Hypertension (OR = 3.72) is also a contributing factor to falls in elderly residents of nursing homes, with over half of middle-aged and elderly people in China suffering from the condition. Hypertension, combined with a decrease in blood pressure regulation ability, the presence of other diseases, and the use of antihypertensive drugs, can lead to non-physiological changes in blood pressure fluctuation amplitude and frequency, making individuals more susceptible to dizziness and increasing the risk of falls [30, 31], which is consistent with the results of a study by Zhang Di [32]. Weiner [33] found that elderly individuals taking psychotropic drugs have a 2.4 times higher risk of falls compared to those who do not take these medications. Multiple guidelines [34, 35] have pointed out that sedatives and hypnotics can increase the risk of falls, which is consistent with the results of this study. Sedatives and hypnotics (OR = 2.36) are a risk factor for falls in elderly residents of nursing homes. Therefore, Medical and nursing staff in elderly care institutions should pay attention to the treatment of various chronic diseases in elderly residents, take medications on time and use medications reasonably, and strengthen the observation of medication side effects. For elderly individuals with visual impairment, early detection and treatment of diseases that cause visual impairment should be carried out, or the use of reading glasses to correct vision should be considered.

#### 4.2.3 Other factors

The results of this study showed that fear of falling, cognitive status, lack of self-awareness of abilities, walking aids, and frequent reminders to prevent falls are risk factors for falls in older adults in long-term care facilities. A study [36] found that older adults who fear falling have a 4.14 times higher risk of falling than those who do not. Fear of falling is an emotional and psychological disorder closely related to falling,which is often manifested as continuous worry about falling in daily life and avoidance of activities within the capacity. Declining cognitive abilities can affect the perception and coping abilities of older adults to the outside world, thereby increasing the risk of falls [37], which is consistent with the findings reported by scholars such as Sun Xiaoya [38]. Older adults with lower activities of daily living and poorer motor function are more likely to fall [39]. In addition, older adults’ lack of self-awareness of abilities includes: overconfidence, lack of protection when engaging in activities such as getting out of bed, reaching for high objects, or hanging clothes, which can lead to falls, or even if they do fall, they are unwilling to seek help from others; inadequate awareness of fall prevention, not realizing the serious consequences that falls may cause [13]. This study showed that walking aids (OR = 1.98) are a contributing factor to falls, which is consistent with the findings of Gell [40]. Improper use of walking aids can increase the risk of falls [41]. Regular reminders from nursing staff to prevent falls have a positive impact on reducing falls in older adults. Therefore, for older adults in long-term care facilities, their activities of daily living should be regularly assessed, and they should be encouraged to engage in appropriate activities to improve their abilities. Regular health education lectures on fall prevention should also be provided to enhance their awareness of fall prevention, while guiding them on the correct use of walking aids.

### 4.3 Limitations

Although this study included 11 provinces, municipalities, and autonomous regions in China, there is limited literature on the factors influencing falls in older adults in Chinese long-term care facilities. Among all retrieved literature, there were more cross-sectional studies with a local focus, while the number of cohort studies and case-control studies was extremely small, with small sample sizes and inadequate coverage. There were significant differences in sample size among the included studies, with the largest sample size being 683 cases and the smallest being 74 cases, leading to some inconsistencies. Additionally, the study results may have been affected by objective factors such as the source of literature and the focus of the research. Therefore, it is necessary to conduct higher quality case-control and cohort studies on falls in older adults in Chinese long-term care facilities, to provide a basis for fall prevention strategies in this population.

## 5 Conclusion

This study showed that the incidence of falls among elderly residents in Chinese nursing homes was relatively high. The main contributing factors to falls among elderly residents in Chinese nursing homes included age, gender, visual impairment, sedative-hypnotics use, fear of falling, hypertension, static balance, having three or more chronic diseases, cognitive status, walking aids, fall-related chronic diseases, self-awareness of abilities, and being frequently reminded to prevent falls. Medical and nursing staff in elderly care institutions should pay attention to these factors and conduct timely screening and interventions to reduce the incidence of falls among elderly residents and avoid the negative impact of falls on their quality of life.

## Supporting information

S1 Checklist (DOCX)

S1 File Search strategy. (DOCX)
