# Supplementary material for: Influencing factors of falls among older adults in Chinese retirement institutions: A systematic review and meta-analysis
Source: PLoS One. 2023 Dec 27;18(12):e0296348. doi: 10.1371/journal.pone.0296348 (PMC10752530; doi:10.1371/journal.pone.0296348)

**Search Strategy**

**CBM：238篇**


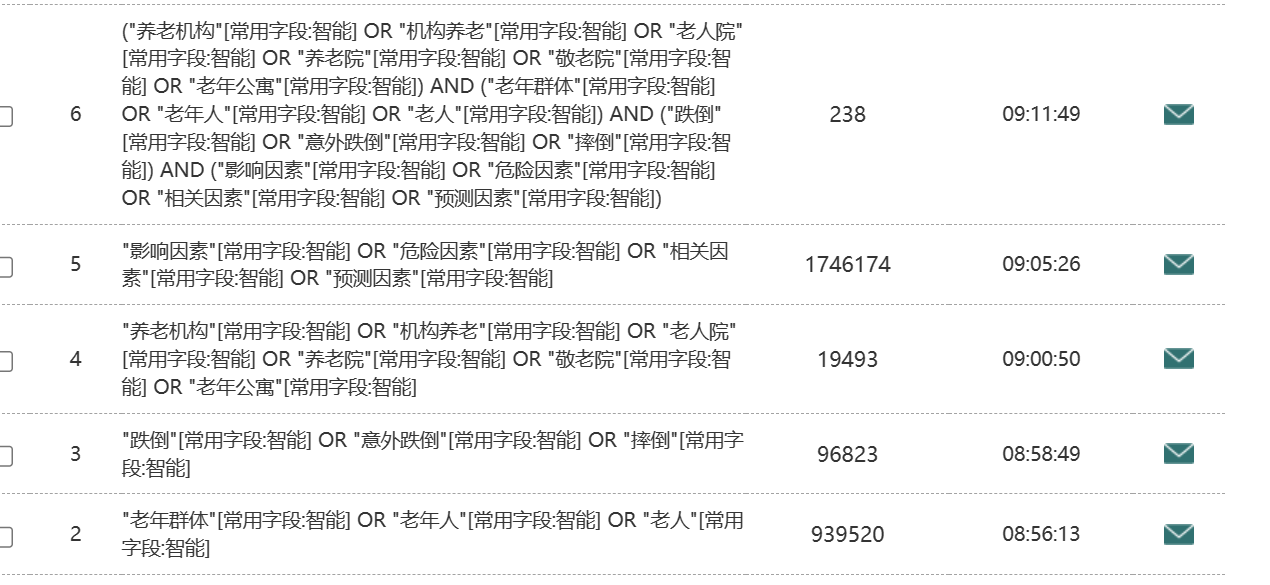


**Pubmed：562篇**


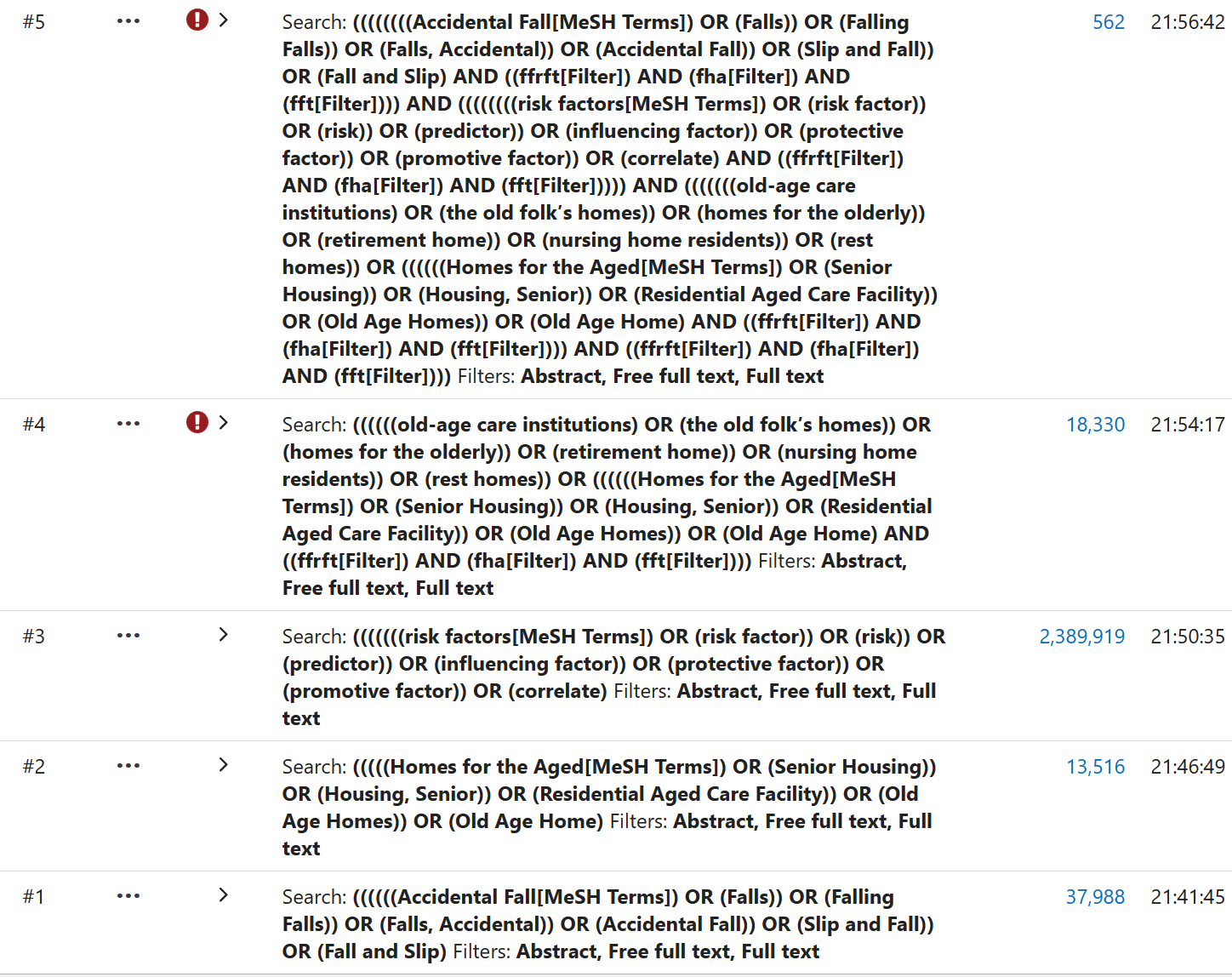


**WEB OF SCIENCE: 617篇**


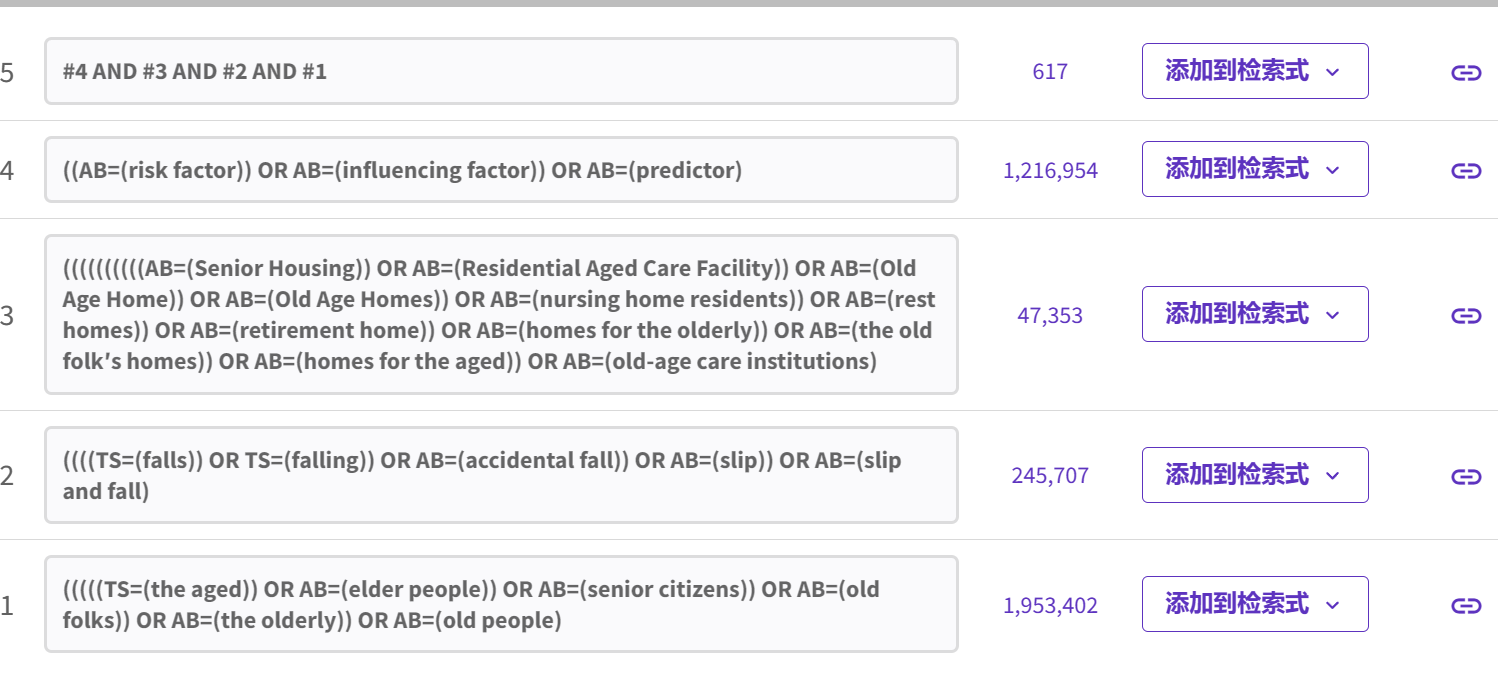


**Embase：99篇**


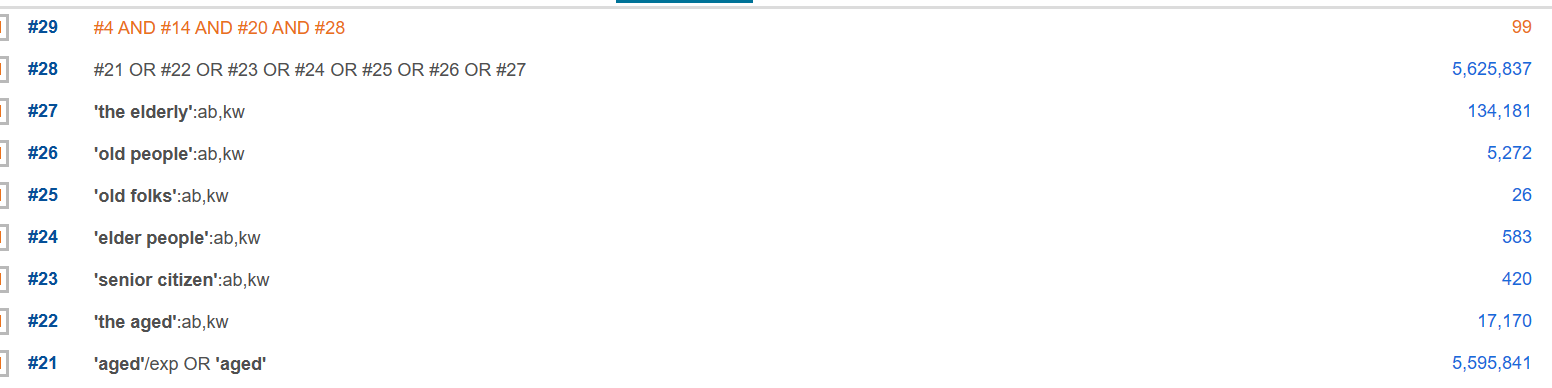


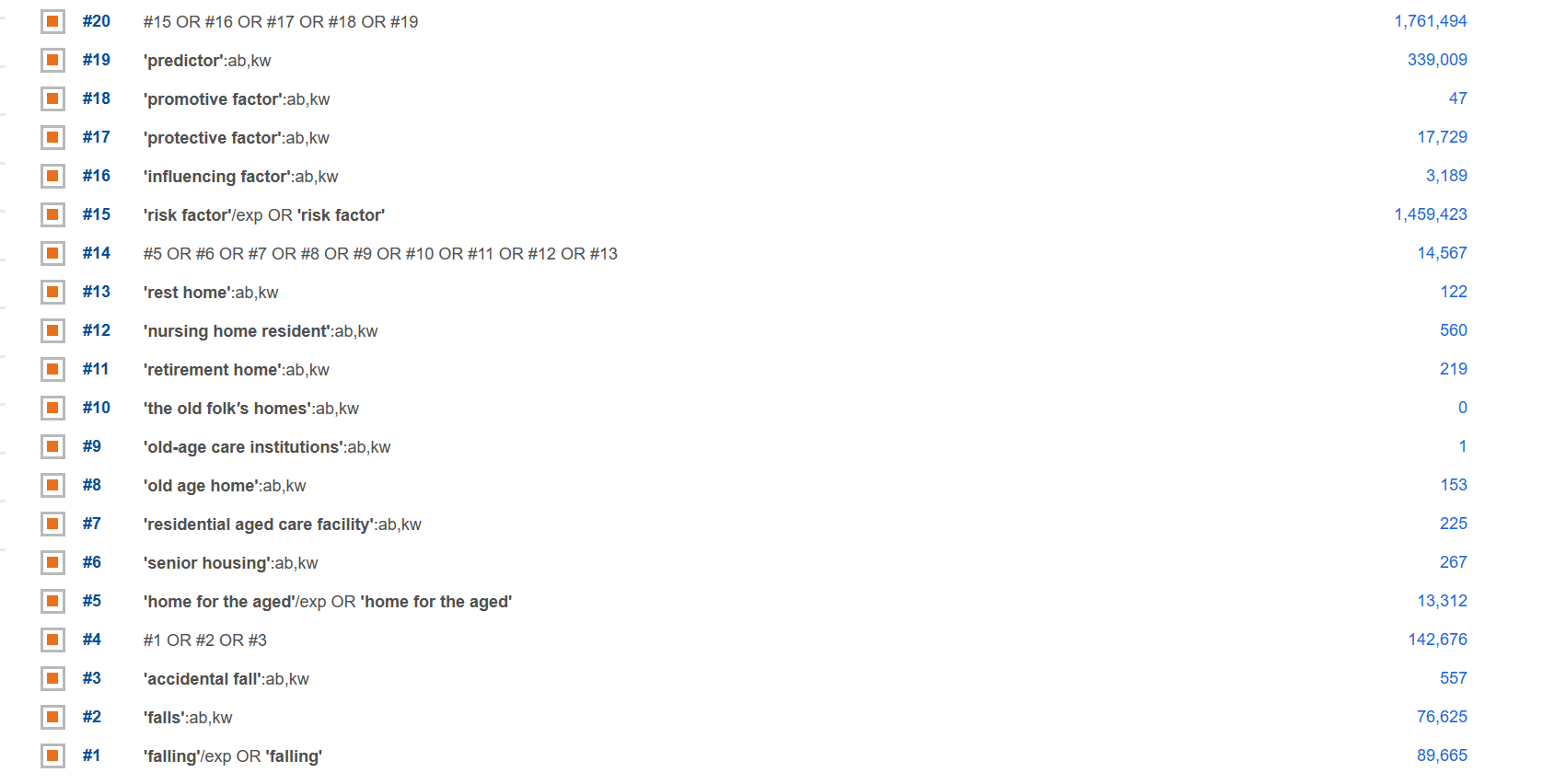


**The Cochrane library：301篇**


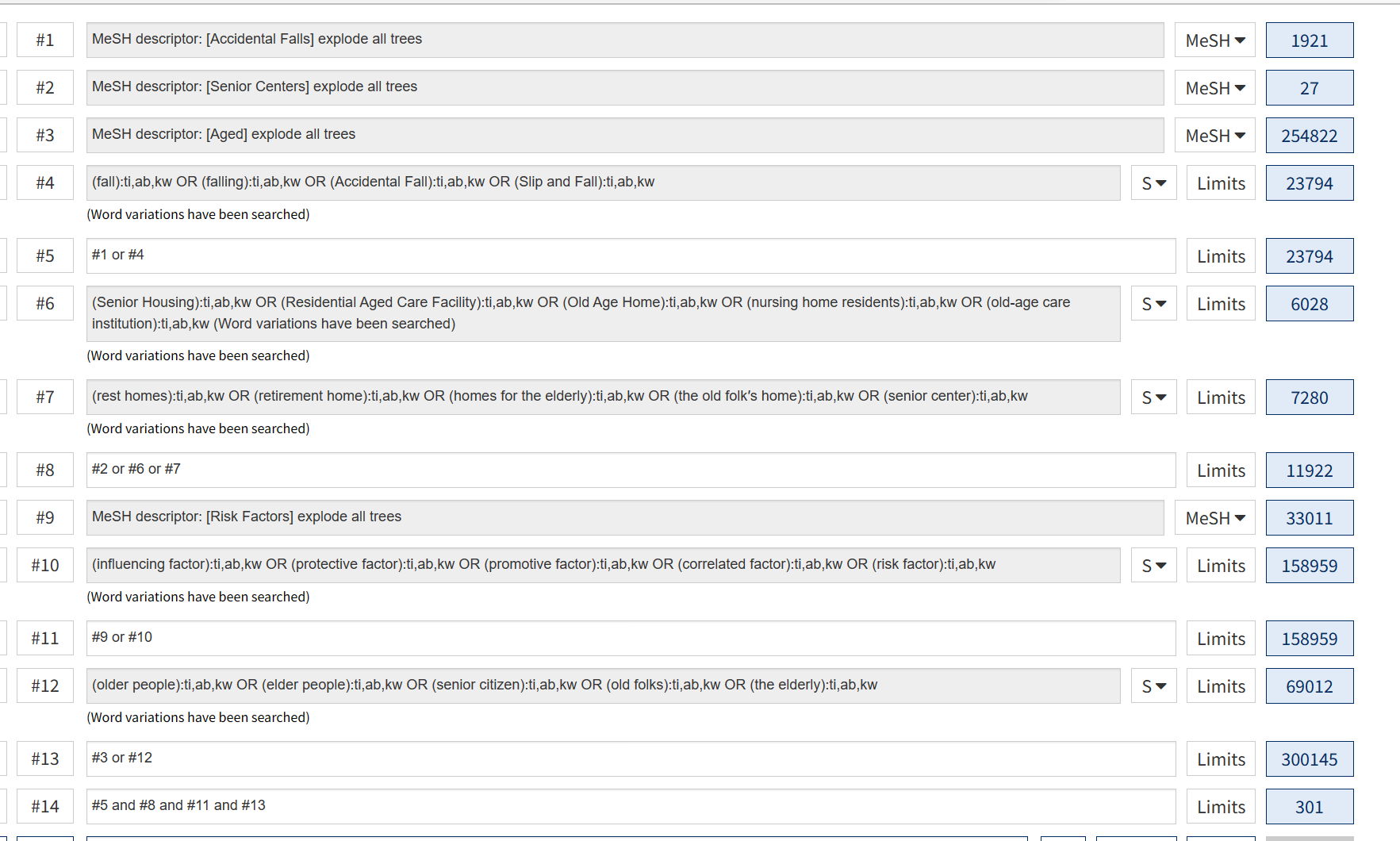


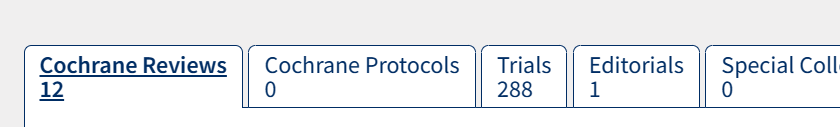


**知网(CNKI)：119**


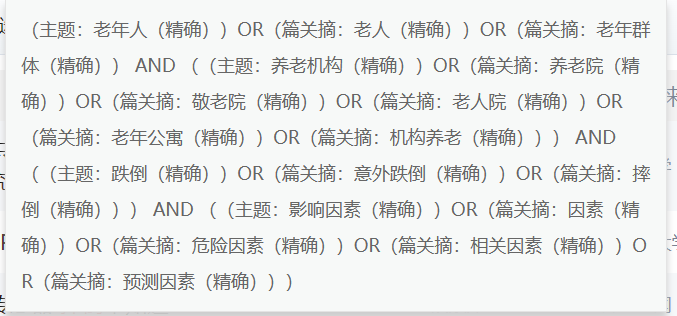


**万方(WangFan)：91篇**


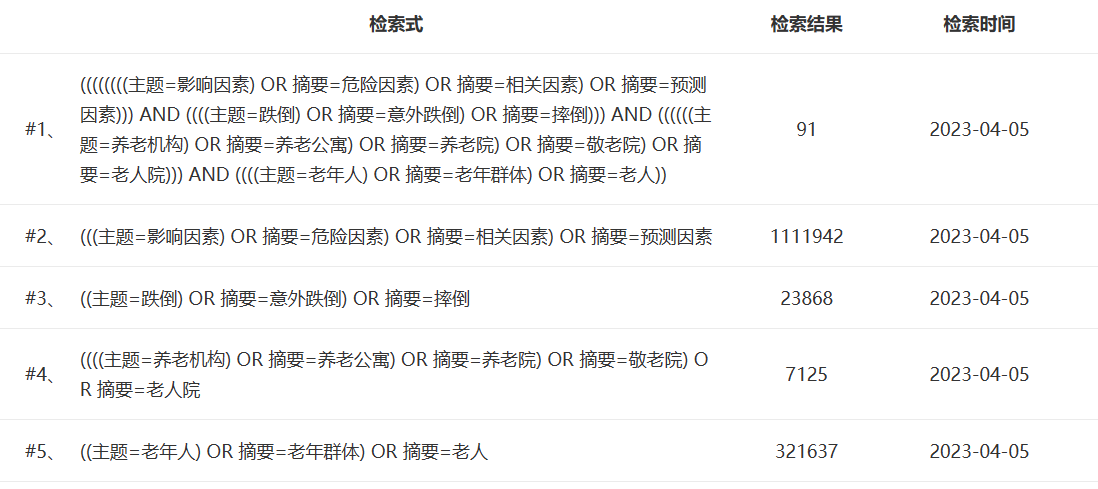


**维普(VIP)：158篇**


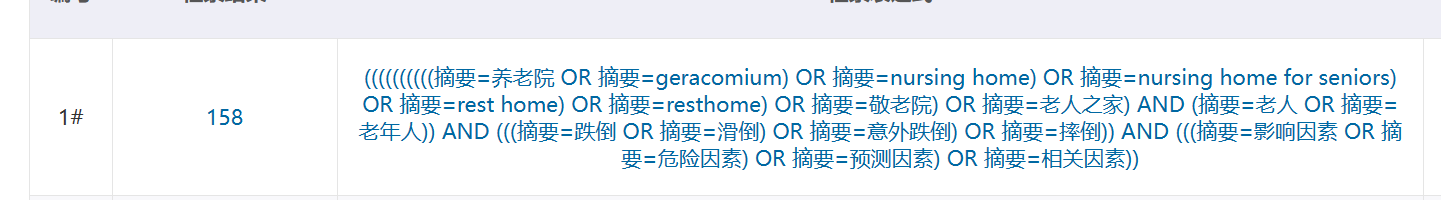

Supplement: S1 File — (DOCX) [file pone.0296348.s002.docx]
